# Senior surgeons as role models in the operating theatre: a thematic analysis through the lens of aristotelian ethics

**DOI:** 10.1186/s12909-022-03921-7

**Published:** 2022-11-30

**Authors:** Mirana Leung-Tack, Divya Khanna, June Jones, Ross O. C. Elledge

**Affiliations:** 1 College of Medical and Dental Sciences, University of Birmingham, Birmingham, UK; 2 Faculty of Health, Social Care and Medicine, Edge Hill University, Ormskirk, UK; 3 University Hospitals Birmingham NHS Foundation Trust, Birmingham, UK

**Keywords:** Ethics, Virtue ethics, Character ethics, Surgical education, Surgical training

## Abstract

**Background:**

Surgeons are commonly evaluated by surgical skills and outcomes rather than their character traits. We sought to examine role model behaviours of senior surgeons through the lens of Aristotelian (virtue) ethics.

**Methods:**

Semi-structured focus group interviews were undertaken of anaesthetic trainees at a large university hospital NHS Foundation Trust and transcripts were subjected to thematic analysis to yield themes and subthemes. Participation of the trainees was entirely voluntary and focus groups were conducted using Zoom™.

**Results:**

The overarching themes identified were ‘Teamwork makes the dream work’, ‘Captain of the ship’ and ‘Strong foundations’.

**Conclusion:**

We hope to take lessons learnt in conjunction with our previous work to help refocus surgical training towards a process of character reformation, rather than simply imparting technical skills to trainees.

## Background

Surgeons are often evaluated by their surgical skills rather than their personal character traits by their trainers, peers and ultimately their patients. The outcomes they achieve and their ability to adhere to the rules set out by employers and regulatory bodies often determine external perceptions of success or failure. Many involved in postgraduate medical and surgical education are now looking deeper at the underlying characters driving these behaviours. There is a growing recognition that surgical training should be a true character reformation as opposed to simply imparting a series of practical skills to trainees [1–3]. Indeed the new surgical curriculum introduced by the Intercollegiate Surgical Curriculum Programme (ISCP) in August 2021 would appear to place increasing emphasis on a move away from competency-based training towards “holistic professional judgement” [4]. The concept of *phronesis* or practical wisdom is particularly useful here, implying good judgment and excellence of character [5, 6]. Whilst this concept has been applied to education in both nursing and primary care [7, 8], surgical training remains very much driven by outcomes exemplified by the work-based assessment system that Aristotle might have termed *techne* in distinction [9].

Senior surgeons who are skilled trainers will enable their trainees to develop robust and dependable clinical judgment by creating conditions which enable trainees to acquire clinical experience and contextual awareness [10]. We have previously highlighted the extremes of poor character in surgeons ‘fallen from grace’ facing fitness to practice hearings through the lens of virtue ethics [11]. With the knowledge that positive character attributes can be reinforced through habituation and role modeling, we sought to explore senior surgeon character and behaviour within the walls of the operating theatre at a large teaching hospital, as perceived by other professionals.

## Methods

Anaesthetic trainees at a large teaching hospital were invited to participate in focus group discussions held online using Zoom™ due to restrictions in place as a result of the COVID-19 pandemic. Attendance at the focus groups was open to all anaesthetic trainees holding a National Training Number, regardless of the level of seniority. Anaesthetic trainees were selected purposefully as they work in close proximity to surgeons and rotate regularly between multiple surgical specialties throughout the course of their training. Participation was entirely voluntary and participants were given written information detailing future plans for the dissemination of findings from the study (including an intention to publish as peer-reviewed research), key contacts and the ability to withdraw at any time. All participants were invited to sign a written consent form attesting that they had understood and agreed with the written information provided.

Focus groups were facilitated by the first author (MLT), an undergraduate medical student at the University of Birmingham. Discussions followed a semi-structured interview, utilizing open questions derived from previous work undertaken by the senior authors (JJ and RE) on the applicability of Aristotelian (virtue) ethics to surgical training [3, 11]. There was an anticipation of 3–5 participants per focus group (with the number of groups dependant upon levels of expression of interest), to enable active participation and engagement of all members of each group.

Focus groups lasted for 60 min and were recorded on a secure Zoom™ Cloud platform. Discussions were transcribed automatically with the Zoom™ audio transcriber and checked for accuracy. Transcripts were downloaded and stored on encrypted devices.

**Fig. 1:**
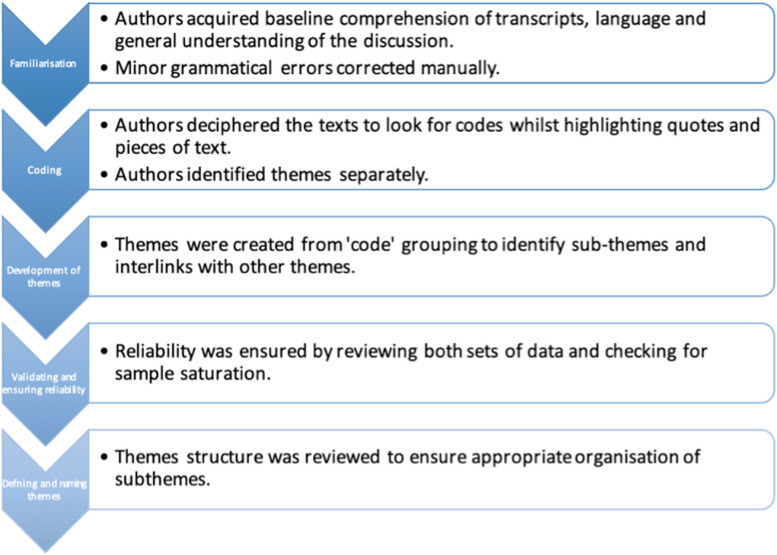
Steps followed in the thematic analysis of focus group transcripts

A deductive thematic analysis of transcripts was carried out independently by two of the authors (MLT and RE) using a modified version of the methodology described by Braun and Clarke [12–14] and adhering to the Consolidated Criteria for Reporting Qualitative Research [15]. This followed the key steps of familiarization, coding, development of themes, validating (and ensuring reliability), defining and name themes (Fig. 1). All three authors then discussed the themes, subthemes and codes identified to identify and refine areas of overlap and disparity.

As participants were NHS staff and participation was voluntary, formal NHS Research Ethics Committee (REC) review was not deemed necessary. This was confirmed following inquiries directed to the Health Research Authority (HRA) and employing NHS Trust Research, Development & Innovation (RD&I) Department. The project was registered with the Clinical Audits and Registries Management Service (CARMS) as a Staff Experience survey.

## Results

Two separate focus groups were convened in June and July 2021. Each focus group comprised three anaesthetic trainees for a total of six participants, spanning first to final year trainees. Four of the participants identified as female and two as male.

Table 1 demonstrates themes, subthemes and codes obtained from the thematic analysis conducted by the authors (MLT and RE), with the final themes being agreed in conjunction with the remaining author (JJ) who supervised and critically appraised the manuscript. The main themes identified were labeled ‘Teamwork makes the dream work’, ‘Captain of the ship’ and ‘Strong foundations’.

**Table 1:** Thematic analysis with overarching themes, subthemes and codes

Overarching themes	Subthemes	Codes
Teamwork makes the dream work	Communications	body language; rudeness; curt; blunt; aggressive expression; (lack of) interdisciplinary communication; damaged rapport; politeness; courtesy; permission; dysfunctional rapport; effective communicator
	Valuing others	different expectations, recognition of; (lack of) cohesion; mismatch of expectations and reality; mutual understandning of other team members; insight; appreciation of time, skillset; complimenting performance; interdisciplinary respect; acknowledgement of worth; overestimating importance of own specialty; dismissiveness; ignorance; snobbish; omitting/ignoring vs. recognizing colleagues’ expertise; demeaning; undermining
	Team player	sharing the load; lack of involvement; trust issues with juniors; equal consideration; chaos; wider teamwork; (lack of) understanding for issues/challenges of others; tribalism; cohesiveness; interdisciplinary teamwork; (un)healthy relationships; (un)motivated team; laying the groundwork for good teamwork when critical; “survival of the fittest” approach; differences in emphasis on teamwork
Captain of the ship	Taking ownership	ownership of the situation; reluctance/willingness to communicate with patients/colleagues; senior mistakes and learning as a team; reputation; leadership; hypocrisy; shirking/assuming responsibility
	Going the extra mile	holistic care; (shirking) responsibility; reliable; need to normalize exemplary behaviours; training in non-technical skills; willingness to teach/educate
	The god complex	snide; snappy; autocratic; dictatorial; profanity; condescending; prestige protecting unacceptable behavior to be tolerated; undermining; imposing agenda; demeaning; embedded and accepted immoral character/actions; abuse of power; power plays; patronizing; adopting “surgical persona”
Strong foundations	The happy workplace	obstructive behavior; tense atmosphere; creating a positive work environment; kindness; reassurance; inclusiveness; joint responsibility; personal connections; building long-term working relationships; bitter relations; good working environment and culture; perpetuating negative/positive working environments
	The flat hierarchy	flattening hierarchies; enabling issues to be raised; familiarity; closeness; “hands on”; interdisciplinary education; mean/aggressive to juniors; earning approved independence and competence; ability to speak up; steeper surgical hierarchy; gender differences

### Teamwork makes the dream work

Within this theme there were examples of positive and negative character traits exemplified by senior surgeons that spanned subthemes of communication, valuing others and positions as team players.

Poor’, ‘ineffective’ or ‘inappropriate’ were among the negative descriptors ascribed by interviewees to communication by senior surgeons within the operating theatre. There were multiple examples given, with one participant observing how on more than one occasion a senior surgical colleague demonstrated ‘no communication at all in theatre, other than to be negative towards someone or to be derogatory’. This was exemplified in body language and, at times, an absence of common courtesy, for example by “not even choosing to try and remember anyone’s name, sometimes not even making eye contact’. Poor communication in other instances extended to ‘using sort of short, snappy and dismissive comments.

By contrast, the ability to recognize the respective skillsets and contributions of other staff within the operating theatre (and value the contributions of these) was recognized by interviewees as a hallmark of excellence in senior surgeons’ role modeling behaviours. The trainees cited the ability of some surgeons to ‘acknowledge that each person [had] their own specific role and that they [were] the expert in that specific role.’ ‘Tribalism’ and the inability of consultant surgeons to perceive value outside of their own remit was observed to lead to a breakdown of trust and shifting of blame, with one interviewee citing an example where a consultant ‘made me cry, this was when I was a foundation doctor for something that wasn’t even my fault, it was a misunderstanding and he just absolutely let loose on me.’ This lack of trust was noted to lead to an undermining of team contributions and dysfunctional interpersonal relationships to the detriment of patient care, with one interviewee noting that ‘it’s very difficult to build up a rapport if someone is obviously not interested in your views or in your presence.’ Fostering long-term stable relationships with colleagues across specialties by contrast was noted to be key to excellence in care.

This loss of trust could in turn lead to a breakdown in cohesion. One trainee observed that if ‘the dynamic between the surgical team and the rest of the theatre team isn’t very cohesive, nobody is really talking out loud, everybody is talking under tones to each other.’ This at best made for unhappy working environments, but at worst has the potential to be dangerous, with one interviewee astutely observing that in a good team with strong foundations ‘when the big case happens that is the life threatening one where you actually do need the good teamwork […] it’s already set up and so you’re not struggling to try and play catch up.’

### Captain of the ship

A point of contention raised was responsibility and effective delegation of duties, balanced with taking ownership of situations. For instance, consultant surgeons whose behaviours generated ‘discord between who’s responsible for communication with the family or with the patient afterwards’ may be a source of friction within the team. Interviewees discussed a noticeable lack of personal involvement from some consultant surgeons to communicate with other team members or with patient’s relatives. Other observations of negative traits were the reluctance of some surgeons to discuss plans with the wider team with one interviewee observing that surgeons may be inclined to ‘come and dictate what their plan is and leave without discussing it.’ By contrast, exemplary behaviours including a willingness to admit personal mistakes to enable team learning, putting patient care before personal reputation and a willingness to communicate effectively with patients and colleagues alike, drawing on everyone’s input in any given situation.

Excellent surgeons appeared to be going the extra mile and offering reliable and dependable leadership and a willingness to teach and educate everyone in the team. They offered holistic care to their patients that went beyond the simple delivery of an operation, often taking the time to train themselves and their trainees formally in non-technical skills, recognizing the importance of these in creating harmonious working environments. There was a discrepancy identified in this respect between surgery and anaesthetics, whereby in the latter ‘other goals such as non-technical skills, team-working, communication [are] really prized and considered almost on par with your ability to anaesthetize’, if not more important.

The god complex was a theme identified in previous work [10] and was exemplified by senior surgeons described as being ‘snide’, ‘snappy’, ‘autocratic’, ‘condescending’, ‘demeaning’ and ‘patronising’. Surgeons who demonstrated these behaviours were seen to adopt a ‘surgical persona’, imposing a strong agenda on teams, with embedded and accepted poor behaviours being perpetuated by a protective veneer of prestige within the hospital. At the extremes of poor behaviour from senior surgeons, the trainees commented that ‘when actually [good behaviour] should just be standard for everyone, it seems the threshold for being “nice” is lower for consultant surgeons, overshadowed by their stereotype.’ This surgeon stereotype was perceived as allowing consultant surgeons to act inconsiderate of others, ‘shouting and ranting about the kit, ended up throwing the kit across the room […] it’s just ridiculous it’s not behaviour you would ever expect in any other environment and somehow that’s accepted and, at the time we all just carried on no one challenged him.’ This accepted stereotype unfortunately allows others to accept expected behaviours. ‘People just accepted [his behaviour] - “well it’s just Mr So-and-so, and this is how he is, it’s part of his job’” […] It was accepted that he didn’t bother to learn any junior’s names, that he would just make names up if he wanted you to do something.’ There was a recognition that this behaviour may come from an ‘old school attitude, which is still there.’

Having a ‘surgical personality’ was not perceived as a compliment. This appeared to stem from the stereotype of the surgeon with a career-driven personality, a survival instinct, a competitive and individualistic attitude to survive a tough hierarchy. ‘They develop different behaviour traits’ one respondent stated, ‘because they have to try and fit in.’ Another observed, ‘surgeons are very, very forceful or behave really strong because they’ve had it their way […] they might have been like that to begin with, and the specialty drew their personality towards it, or maybe they just develop these behavioural traits in order to survive.’

### Strong foundations

Positive character and good moral conduct in leadership and team-working was seen as enabling a positive working atmosphere and happy workplace that excellent surgeons developed ‘over decades’ to prevent a ‘vicious circle of bad habits.’ A working environment ‘where people are so frightened or […] disillusioned’ and ‘wouldn’t really dare to speak up if you’re doing [the] obviously wrong procedure, or if someone or something is not right’ was clearly regarded as potentially detrimental to patient care by the interviewees.

Examples of senior surgeons developing flawed, tense and confrontational work environments included one cited by an interviewee of a consultant surgeon “being really mean, racist and sexist about his colleagues around him, like his registrar who wasn’t there at the time, and it just made me think that I just don’t want to do anything wrong in front of [him] so that [he is] not bad mouthing me to everyone around [him].’ Another example was cited of a consultant ‘[hitting] his registrar’s knuckles because they got in the way […] he had done it three times and […] I thought it was completely unacceptable.’ The trainees recognized that junior surgical colleagues may have difficulty responding in such situations as ‘because of the hierarchy […] it’s really difficult for them to say, please stop doing that to me […] or feel like it wasn’t going to do their career any good […] and so just keep quiet.’

Overconfidence among senior surgeons was cited by interviewees as leading them to have unrealistic expectations of their own abilities. ‘They say, “I’m going to take half an hour to close” and then an hour later they’re still closing and it’s just a tiny example […] but if a surgeon keep doing that then it ruins that trust.’

Creating a positive work environment was liable to come about through consultant surgeons demonstrating particular qualities that interviewees felt included kindness, reassurance and inclusivity. Surgeons who invested in building long-term working relationships, establishing shared responsibility for patient care and nurturing good connections with team members developed strong teams and were positive role models.

A point that interviewees often returned to was the need to achieve a flat hierarchy, despite acknowledging that ‘there needs to be a hierarchy.’ This hierarchy manifests in the theatre environment in different ways. Personal introductions might ‘set the bar as to where the hierarchy is’, but this might be reinforced by behaviour, with one interviewee commenting that poor role models might ‘feel like they’re above themselves to come to a meeting about the case that we’re going to do, and it sort of implies […] they’re just going to come and do their thing, and all of us are just there to facilitate it.’ Further examples of poor behaviours cited included surgeons who ‘don’t take on board what [colleagues] are saying’, ‘don’t listen’ and who failed to give due regard to opinions of colleagues. One interviewee observed that ‘toxic behaviours are born out of some need to maintain a hierarchy […] otherwise it would be chaos […] but there needs to be a point where everything is flattened out and then everyone has an equal voice.’

An inability to raise issues was cited as concerning, but conversely there was a recognition that ‘the best list […] is where the whole theatre is able to speak up’ with excellent surgeons saying ‘if you see something to help keep me safe and the patient safe, then please speak up because no question or comment will be looked down upon.’

## Discussion

In this project we interviewed two focus groups of anaesthetic trainees working in the theatre environment. In order not to bias the discussion, open questions were asked to establish their opinions and observations regarding both good and bad behaviours and character traits of consultant surgeons. MLT was specifically chosen as the facilitator as it was felt that her position as a non-threatening undergraduate medical student would enable participants to be open and honest in their interviews. Clearly, bias exists in the selection of any facilitator, as the presence of a medical student will be inclined to elicit a different range of responses to alternatives such as allied health professionals, non-clinical research staff and others.

The conversation appeared disproportionately biased towards the negative attributes observed, but it is important to acknowledge that as one respondent stated: ‘the stories […] shared, […] the bad ones, have been two in number and people described as excellent is 10 times that amount of people […] but the problem is that these things are stereotypes for a reason and the bad ones stand out and get remembered. And the ones who just come to work and do an excellent job aren’t remembered as easily as the ones that made everyone cry unfortunately’. This echoes the observations of a previous work regarding the analysis of Medical Practitioner Tribunal Services (MPTS) hearings of consultant surgeons, where only a small proportion, 0.001% of registered surgeons, faced fitness to practice hearings [11].

Much of the positive drive to change in surgical culture and education can arguably be said to constitute attempts at behavior modification, as opposed to targeting underlying character traits, internal drivers and the thought processes behind such behaviours. Commendable examples include the focus on lessons learnt from the aviation industry in applying human factors training to operating theatres [16] and the Civility Saves Lives movement [17, 18]. Brennan discusses behaviours in the operating theatre and suggests that any negative interaction in the operating theatre can have a detriment on patient safety [19]. Civility Saves Lives argues that if consultant surgeons were more approachable, this would make the multi-disciplinary team more effective and thus lead to improved patient outcomes [18].

One could argue that behaviour modification could help improve surgical team performance in the operating theatre. However, behaviour modification is too superficial an approach in the authors’ view. According to Aristotle [5], *akrasia* refers to a person with enough self-mastery to ‘do the right thing’. The Aristotelian principle of virtuous life (*arête*) argues that life is only worth living if the supreme good is pursued by humans to achieve *eudaimonia* (‘flourishing’). This means that we should not have to direct consultant surgeons (or surgical trainees) to behave in a particular manner (e.g. common courtesy, flattened hierarchies, etc.) through behaviour modification. Rather, good intentions should be a reflection of their genuine character traits, instilled through positive role modeling and habituation in the formative process of surgical training.

It is recognized that due attention should be given to so-called ‘softer’ skills in surgical training, with such recognition being enshrined in the validated Non-Technical Skills for Surgeons (NOTSS) behaviour assessment tool [20, 21]. In the interviews, the pursuit of power and the development of a god complex and surgical persona seemed to be overriding character flaws manifesting as a result for the need to maintain hierarchy in the theatre environment. Aristotle promoted the concept of a golden mean whereby virtues and vices were not opposites but rather excesses or deficiencies of particular aspects of character [5, 6, 22]. Modesty (*aidos*) therefore lies between shamelessness (*anaischuntia*) and shyness (*cataplexis*), honesty (*aletheia*) between boastfulness (*alazoneia*) and understatement (*eironeia*), friendliness (*philia*) between cantankerousness (*duskolia*) and obsequiousness (*areskeia*), and so on. Redolent of this is the notion that a senior surgeon must be a leader in the operating theatre, but one who empowers others and draws the best from his or her team, rather than pushing a single-minded agenda. A failure to recognize the importance of temperance (*sophrosune*) leads to cases such as Ian Paterson [23, 24] on an individual scale and Mid Staffordshire NHS Foundation Trust [25, 26] on an institutional one, where people feel unable to speak up in the face of overbearing characters, a key point raised throughout these interviews. This is not to say that a flattened hierarchy is always the ideal however, and the ability of leaders to be sure of their decisions, exhibit well-founded self-confidence and to assume control when required is no less important to patient safety than the ability of juniors to speak up [27].

Through the lens of Aristotelian (virtue) ethics, a ‘good’ surgeon is not just one who is technically brilliant and achieves good outcomes ‘they are also a surgeon whose character aims for the mean between excess and deficiency in the moral choices inherent in a career in surgery [11].’ We can question whether the surgical training surgeons go through reinforces certain character traits and extremes of behaviours or whether these traits are required to successfully thrive through surgical training. The Aristotelian ethical approach argues that one needs to fundamentally believe they are genuinely doing ‘good’ for the team and the patient to improve patient outcomes. If behaviour is a manifestation of character, we argue that ‘good’ character traits should be instilled during training, which should be a comprehensive character reformation rather than merely imparting skills, not least through positive role modeling. Above all, we need to move away from teaching surgeons to simply ‘do’, and focus on teaching them to ‘be’, making them the kind of people worthy of holding in high regard, rather than simply imbuing them with a skillset. This seems to be a growing feeling more widely within surgical education, as exemplified by the changes to the new surgical curriculum and methods of assessment by ISCP, moving trainees away from pure competency-based assessment [4].

Small numbers of participants in focus groups such as these is standard. Limitations can be identified however including the possibility of “response bias” due to such a small number of trainees potentially feeling “pressured” in responding a particular way by peers or expectations. The use of MLT as a facilitator was planned to mitigate against bias and encourage respondents to be candid. One main limitation of this project was the pressures of the COVID-19 pandemic at the time of data collection. Initially we planned to carry out the interviews with Operating Department Practitioners (ODPs) and theatre nursing staff, as well as junior surgical trainees. We also had logistic issues to obtain a large focus group as all interviewees were working on different shifts, impacted in part by the ongoing response to the COVID-19 pandemic at one of the hardest hit NHS Trusts in the country. Despite this, having two different focus groups of interviewees from the same specialty enabled sampling to saturation exhibited by the two transcripts, whilst also validating the themes found between the two separate groups. We hope to continue this project with other staff groups in the future to obtain a more balanced view of our findings.

## Data Availability

The datasets used and/or analysed during the current study are available from the corresponding author on reasonable request.

## Declarations

All methods were carried out in accordance with relevant regulations and protocol was approved by University Hospitals Birmingham NHS Foundation Trust (CARMS-16941). Informed consent was obtained from all subjects and participation was entirely voluntary.
